# Zearalenone exposure differentially affects the ovarian proteome in pre-pubertal gilts during thermal neutral and heat stress conditions

**DOI:** 10.1093/jas/skae115

**Published:** 2024-04-26

**Authors:** Crystal M Roach, Edith J Mayorga, Lance H Baumgard, Jason W Ross, Aileen F Keating

**Affiliations:** Department of Animal Science, Iowa State University, Ames, IA 50011, USA; Department of Animal Science, Iowa State University, Ames, IA 50011, USA; Department of Animal Science, Iowa State University, Ames, IA 50011, USA; Department of Animal Science, Iowa State University, Ames, IA 50011, USA; Department of Animal Science, Iowa State University, Ames, IA 50011, USA

**Keywords:** heat stress, proteome, pigs, reproduction, zearalenone

## Abstract

Zearalenone (ZEN), a nonsteroidal estrogenic mycotoxin, causes endocrine disruption and porcine reproductive dysfunction. Heat stress (HS) occurs when exogenous and metabolic heat accumulation exceeds heat dissipation. Independently, HS and ZEN both compromise swine reproduction; thus, the hypothesis investigated was two-pronged: that ZEN exposure would alter the ovarian proteome and that these effects would differ in thermal neutral (TN) and HS pigs. Pre-pubertal gilts (*n* = 38) were fed ad libitum and assigned to either (TN: 21.0 ± 0.1 °C) or HS (12 h cyclic temperatures of 35.0 ± 0.2 °C and 32.2 ± 0.1 °C). Within the TN group, a subset of pigs were pair-fed (PF) to the amount of feed that the HS gilts consumed to eliminate the confounding effects of dissimilar nutrient intake. All gilts orally received a vehicle control (CT) or ZEN (40 μg/kg/BW) resulting in six treatment groups: thermoneutral (TN) vehicle control (TC; *n* = 6); TN ZEN (TZ; *n* = 6); PF vehicle control (PC; *n* = 6); PF ZEN (PZ; *n* = 6); HS vehicle control (HC; *n* = 7); or HS ZEN (HZ; *n* = 7) for 7 d. When compared to the TC pigs, TZ pigs had 45 increased and 39 decreased proteins (*P* ≤* *0.05). In the HZ pigs, 47 proteins were increased and 61 were decreased (*P* ≤* *0.05). Exposure to ZEN during TN conditions altered sec61 translocon complex (40%), rough endoplasmic reticulum membrane (8.2%), and proteasome complex (5.4%), asparagine metabolic process (0.60%), aspartate family amino acid metabolic process (0.14%), and cellular amide metabolic process (0.02%) pathways. During HS, ZEN affected cellular pathways associated with proteasome core complex alpha subunit complex (0.23%), fibrillar collagen trimer (0.14%), proteasome complex (0.05%), and spliceosomal complex (0.03%). Thus, these data identify ovarian pathways altered by ZEN exposure and suggest that the molecular targets of ZEN differ in TN and HS pigs.

## Teaser

This study examined the role of zearalenone-induced ovarian toxicity and any different effects of ZEN exposure during heat stress in the pre-pubertal pig. Zearalenone impairs ovarian function and molecular targets identified could lead to mitigation strategy development.

## Introduction

Zearalenone (ZEN) is an estrogenic nonsteroidal fungal mycotoxin from the genus *Fusarium* (Shier et al., 2001; Bennett and Klich, 2003; Zinedine et al., 2007) and a food contaminant due to high-temperature resistance during food processing (Bennett et al., 1980). Detectable in wheat bran and wheat germ oil, corn and corn germ oil, and corn by-products (Olsen et al., 1981; EFSA, 2011), ingestion is the primary ZEN exposure route. Absorption of ZEN is rapid (Olsen et al., 1985; Kuiper-Goodman et al., 1987) and the half-life of ZEN in pigs is ~86 h, with absorption estimated at 80% to 85% from the gastrointestinal tract (Biehl et al., 1993).

Zearalenone is an endocrine-disrupting chemical (Metzler et al., 2010; Frizzell et al., 2011), by structurally mimicking endogenous estradiol (E_2_)_,_ and binding to the estrogen receptor (Shier et al., 2001); a molecular scenario that inhibits the estrogen response element-mediated regulation of gene transcription (Bennett and Klich, 2003; Minervini and Dell’Aquila, 2008). In vivo, exposure to intraperitoneal ZEN at 7.5 mg/kg/b.w. for 24 h inhibited follicle-stimulating hormone synthesis and secretion, and gilts administrated 10, 20, or 40 μg/g b.w. of ZEN-contaminated feed for 28 d had enlarged uteri (James and Smith, 1982). Further evidence of an estrogenic impact of ZEN was observed by increased reproductive tract weight in female pigs administered 1.1, 2.0, or 3.2 mg/kg b.w of ZEN for 18 d (Jiang et al., 2011).

Biotransformation of ZEN is species-dependent, and secondary metabolites formed may contribute to ZEN’s toxicity (Malekinejad et al., 2006). Metabolism of ZEN occurs by two major biotransformation pathways: phase I reduction and phase II glucuronidation and sulfation. In poultry and cattle, β-ZOL is the primary ZEN metabolite (Joint FAO/WHO Expert Committee on Food Additives, 2000; Malekinejad et al., 2006; Zinedine et al., 2007; Videmann et al., 2008). However, in pigs, α-ZOL is the primary ZEN metabolite and this is responsible for pigs being highly sensitive to ZEN because α-ZOL has a high affinity for the estrogen receptor (Malekinejad et al., 2006). Incidentally, the pig has similar ZEN biotransformation and sensitivity levels as humans (Pillay et al., 2002; Malekinejad et al., 2006; Videmann et al., 2008), which differs from rodents (Malekinejad et al., 2006). A provisional maximum tolerable daily intake for ZEN is 0.2 µg/kg body weight (b.w.) in humans (Joint FAO/WHO Expert Committee on Food Additives, 2000), based on a no observed effect level (NOEL) of 40 µg/kg b.w. in pigs (Joint FAO/WHO Expert Committee on Food Additives, 2000). In gilts, the lowest observed effect levels for the ovary, uterus, and vulva range from 17 to 200 µg/kg b.w. with a NOEL level of 10 µg/kg b.w. (EFSA, 2011), but currently, a provisional maximum tolerable daily intake value for ZEN is not defined in the United States.

Heat stress (HS) also impairs female fertility (Ross et al., 2017); phenotypically manifested as prolonged return to estrus (Love, 1978), reduced embryonic development (Isom et al., 2007; Bertoldo et al., 2010), and impaired pregnancy maintenance (Love et al., 1993; Tast et al., 2002). Ovarian mechanisms of HS-induced infertility include granulosa cell apoptosis (Luo et al., 2016), autophagy (Hale et al., 2017, 2021), reduced corpora lutea diameter and weight (Bidne et al., 2019; Romoser et al., 2022), and altered abundance of insulin-mediated and steroidogenic intracellular signaling (Nteeba et al., 2015), toll-like 4 receptors (Dickson et al., 2018), estrogen sulfotransferase (Dickson et al., 2018) and heat shock proteins (HSP; Seibert et al., 2019). Thus, HS impairs ovarian function and some molecular mechanisms are partially defined.

During HS, basal insulin concentrations increase (Itoh et al., 1998; Wheelock et al., 2010; Pearce et al., 2013), despite a reduction in feed intake (FI). This presents a potential link between HS and an altered response to chemical exposure since experiments in hepatic cells have demonstrated that insulin and glucagon both regulate proteins involved in chemical biotransformation. In cultured hepatocytes, lack of insulin reduced Cytochrome P450 (CYP) isoforms 2B (CYP2B), 3A (CYP3A), and 4A(CYP4A) in response to xenobiotic induction (Woodcroft and Novak, 1999). Insulin also increased hepatic abundance of microsomal epoxide hydrolase (EPHX1) and this was ameliorated by inhibition of phosphatidylinositol 3-kinase (PI3K) or mitogen-activated protein kinase pathways (Kim et al., 2003). Conversely, glucagon decreased both hepatic EPHX1 and CYP isoform 2E1 (CYP2E1) protein abundance (Woodcroft et al., 2002; Kim et al., 2003). The insulin-regulated PI3K pathway also regulates ovarian chemical biotransformation (Bhattacharya and Keating, 2012; Bhattacharya et al., 2012, 2013). Thus, the potential for HS to change the ovarian response to an ovotoxicant is convincing, especially within the context of climate change.

This study investigated ovarian molecular targets of ZEN exposure and evaluated if this response differed in HS pigs. We hypothesized that ZEN exposure would alter the ovarian proteome and that these effects would diverge in thermal neutral (TN) and HS pigs.

## Material and Methods

### Animal and experimental design

All animal procedures were approved by the Institutional Animal Care and Use Committee at Iowa State University. This study utilized tissues collected from a previously described experiment (Roach et al., 2024). Briefly, female crossbred pre-pubertal gilts (61.5 kg ± 0.5; 105 to 115 d of age; *n *= 38) were fed a standard diet formulated to meet all nutritional requirements (National Research Council, 2012). Gilts were exposed to constant TN conditions (21.0 ± 0.1 °C, 66.8% relative humidity) or cyclic HS (35.0 ± 0.2 °C from 0700 to 1900 hours, 42.0% relative humidity and 32.2 ± 0.1 °C from 1900 to 0700 hours, 40.7% relative humidity) for 7 d. Environmental temperatures were selected to simulate summer conditions in the midwestern region of the United States. All animals experienced a 12:12 h light–dark cycle. The TN gilts were further divided into two subgroups which were either ad libitum fed (TN) or were pair fed (PF) to calorically control for the reduction in FI which occurred in the HS gilts, and treatments were assigned as thermoneutral (TN) vehicle control (TC; *n* = 6), TN ZEN (TZ; *n* = 6), PF vehicle control (PC; *n* = 6), PF ZEN (PZ; *n *= 6), HS vehicle control (HC; *n* = 7), HS ZEN (HZ; *n* = 7). The vehicle control and the ZEN (40 µg/kg BW; 0.04 ppm; Z2125, Sigma-Aldrich, Inc. St. Louis, MO) were provided in a 10 g cookie dough bolus at 0700 and 1900 hours. This dosage was based on previous publications in which ovarian effects were observed (Liu et al., 2017) and based on the level of human exposure (Kuiper-Goodman et al., 1987; Joint FAO/WHO Expert Committee on Food Additives, 2000).

## Tissue Collection

Pigs were euthanized on day 7 using captive bolt and exsanguination and one ovary was immediately collected, weighed, and snap-frozen in liquid nitrogen followed by storage at -80°C.

## Ovarian Protein Isolation and Quantification

Whole ovarian tissue was powdered with a mortar and pestle on dry ice. Approximately 100 mg of powdered whole ovarian tissue was weighed and lysed by tissue lysis buffer (200 µL; 50 mM Tris-HCl, 1 mM EDTA, pH 8.5) supplemented with Halt protease and phosphatase inhibitor cocktail (P178442, Thermo Scientific, Waltham, MA). Lysed tissue was homogenized by sonication and incubated on ice for 30 min. Protein lysate was centrifuged at 10,000 rpm for 15 min at 4 °C and supernatant was collected. Protein concentration was quantified using a Pierce BCA Protein Assay Kit (BCA; 23227, Thermo Scientific, Waltham, MA) and spectrophotometry detection.

## Liquid Chromatography-Tandem Mass Spectrometry

Total ovarian protein samples were prepared with a working solution of 50 µg/µL diluted in lysis buffer. Liquid chromatography-tandem mass spectrometry (LC-MS/MS) analysis was performed as previously described (Clark et al., 2019; González-Alvarez et al., 2021) at The Protein Facility of the Iowa State University Office of Biotechnology. Briefly, 50 µg/µL of total protein was digested with trypsin/Lys-C for 16 h, dried and reconstituted in buffer A (47.5 µL; 0.1% formic acid/water). A standard Peptide Retention Time Calibration (PRTC; 25 fmol/µL) was spiked into each sample as an internal control. Protein and PRTC were injected into an LC column and separated by mass spectrometry. Fragmented patterns were compared to MASCOT or Sequest HT theoretical fragmentation patterns for peptide identification. The area of the top three unique peptides per sample was used to identify protein abundance. The PRTC arithmetic mean was used as a normalization factor. The signal intensity was divided by the PRTC arithmetic mean for each peptide. Protein identities were confirmed by three peptides for each protein. Metaboanalyst 4.0 was used for bioinformatics comparison by the Genome Informatics Facility at Iowa State University. Missing value imputation by Singular Value Decomposition method was performed. Values were filtered based on the interquartile range followed by generalized log transformation. Volcano plots depict alterations to proteins within treatments. UniProt identified biological, molecular, and pathway information using KEGG identifiers for each protein.

### Gene ontology analysis and protein-protein interaction web network

Gene Ontology (GO) analysis was conducted using the protein-coding gene classification system Search Tool for the Retrieval of Interacting Genes/Proteins (STRING) and to identify pathways of altered proteins within comparisons. Proteins altered by treatments with a P ≤ 0.05 were compared to the Sus scrofa reference list for pathway classification. The percentage of each category was calculated by dividing the gene hit by the total number of genes. Common ovarian proteins were identified across treatment comparisons and protein-protein interactions/web network was computed using STRING.

## Statistical Analysis

Student’s t-test was used to compare control and treatment with the adjusted *P*-value false discovery rate (FDR) cutoff of 0.05. A fold change threshold of 1 was used to compare the absolute value of change and expression level between control and treatment values. The threshold for proteins deemed significant in the pathway analysis was *P* ≤ 0.05.

## Results

### Effects of ZEN exposure on the whole ovarian proteome in TN, PF, and HS pigs

**Figure 1.**
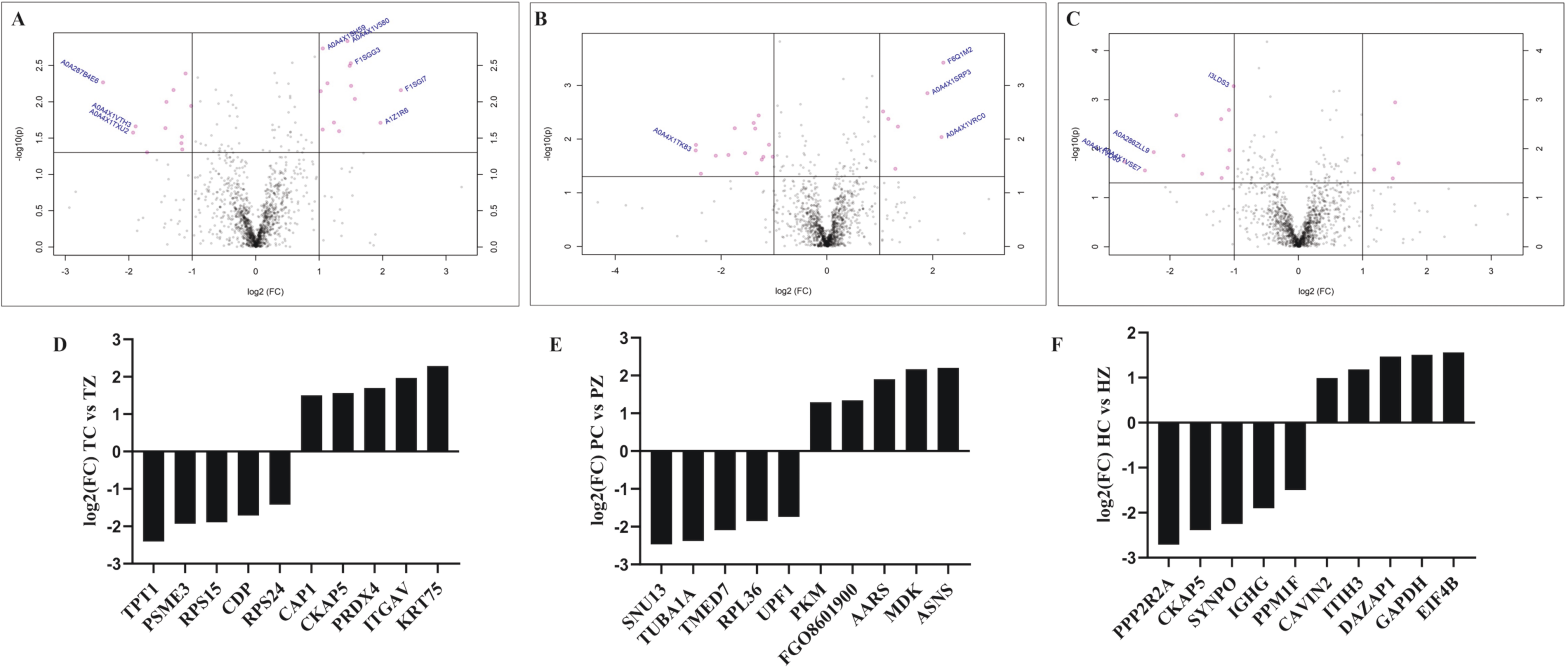
Ovarian proteins altered by ZEN exposure. The volcano plot depicts the comparison between proteins identified in (A) TC vs. TZ, (B) PC vs. PZ, and (C) HC vs. HZ gilt ovaries. The solid horizontal line indicates where P = 0.05, with dots above the line P < 0.05 and dots below having P > 0.05 The solid vertical line indicates log2fold change of < ± 1.0 with dots to the right indicating increased and dots to the left denoting decreased proteins relative to respective control. Bar chart represents the top five increased and decreased proteins per comparison illustrated as fold-change in (D) TC vs TZ, (E) PC vs PZ, and (F) HC vs HZ in pre-pubertal gilts. TC: n = 6; TZ: n = 6; PC: n = 6; PZ: n = 6; HC: n = 7; HZ: n = 7; P ≤ 0.05.

Relative to TN gilts, 84 proteins were altered by ZEN (*P* ≤* *0.05; Figure 1A; Supplementary Table 1), with 45 increased and 39 decreased. Relative to PF gilts, ZEN altered 84 proteins (*P* ≤* *0.05; Figure 1B; Supplementary Table 2) with 45 increased and 39 decreased. While identical numbers of increased and decreased proteins due to ZEN exposure when compared to the control ovaries in each nutritional group were identified, the protein identities differed (Supplementary Tables 1 and 2). Relative to HS gilts, ZEN altered 108 proteins (*P* ≤* *0.05; Figure 1C; Supplementary Table 3) with 47 increased and 61 decreased.

Based on fold-change, the five most reduced ovarian proteins were TPT1, PSME3, RPS15, CDP, and RPS24 and the five most increased by ZEN exposure were CAP1, CKAP5, PRDX4, ITGAV, and KRT75, relative to TC gilts (P ≤ 0.05; Figure 1D). The five ovarian proteins increased to the greatest extent by ZEN in PZ relative to PC gilts were PKM, FGO8601900, AARS, MDK, and ASNS, and the five most decreased proteins were SNU13, TUBA1A, TMED7, RPL36, and UPF1 (P ≤ 0.05; Figure 1E). In HS gilts, the five ovarian proteins increased most by ZEN were CAVIN2, ITIH3, DAZAP1, GAPDH, and EIF4B, and the five most decreased proteins were PPP2R2A, CKAP5, SYNPO, IGHG, and PPM1F (P ≤ 0.05; Figure 1F).

## Functional Classification of Biological Pathways Identified to be Altered in the Ovary by ZEN Exposure

Proteins identified to be different by ZEN (*P* ≤* *0.05) relative to TN, PF, and HS gilts were designated to classification systems based on their ascribed function using STRING GO analysis.

**Figure 2.**
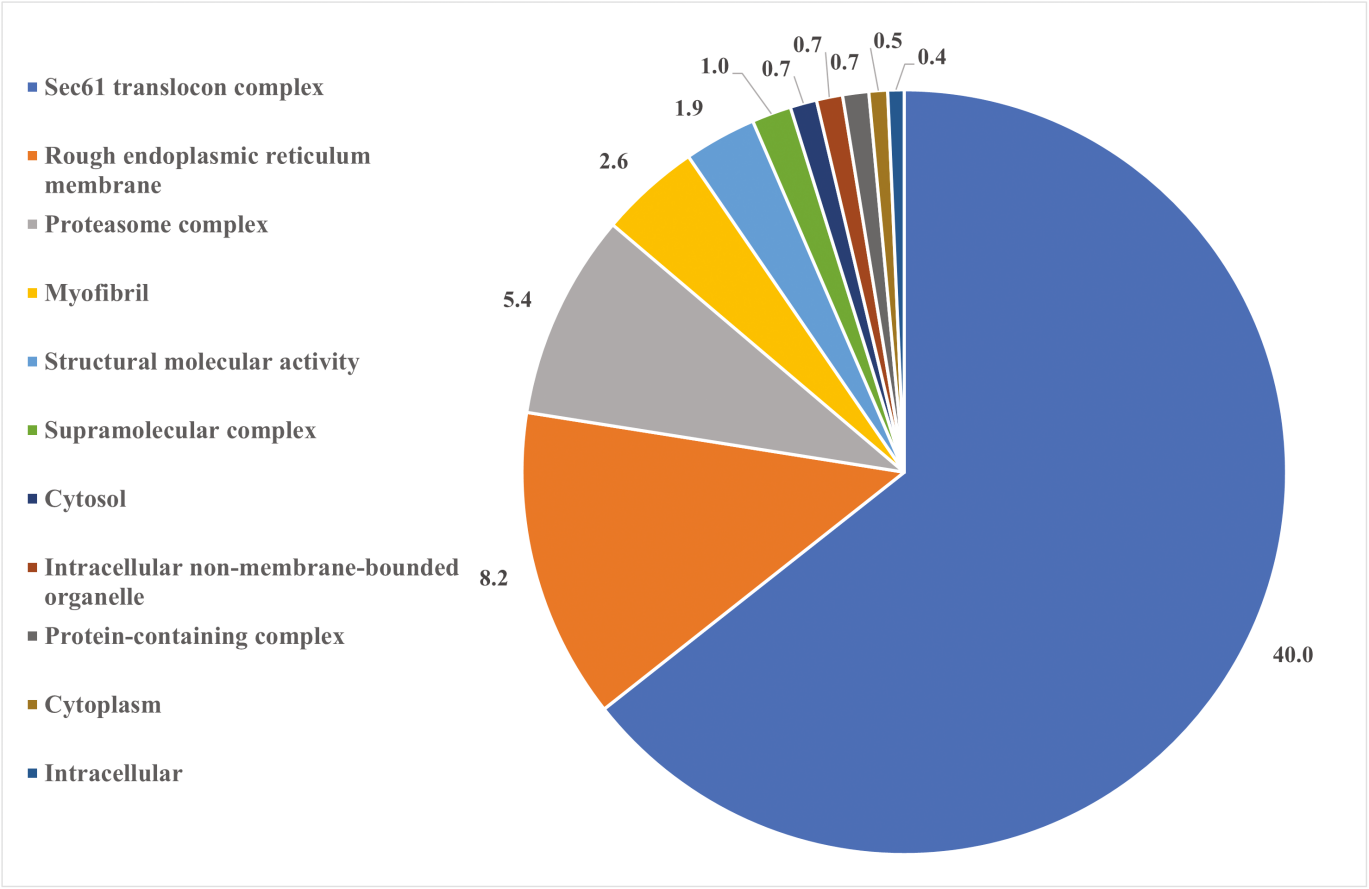
Distribution of biological processes in ovarian proteins altered by ZEN exposure in thermal neutral gilts. Pie chart represents the biological processes in TC vs. TZ pre-pubertal gilts; FDR ≤ 0.05.

**Figure 3.**
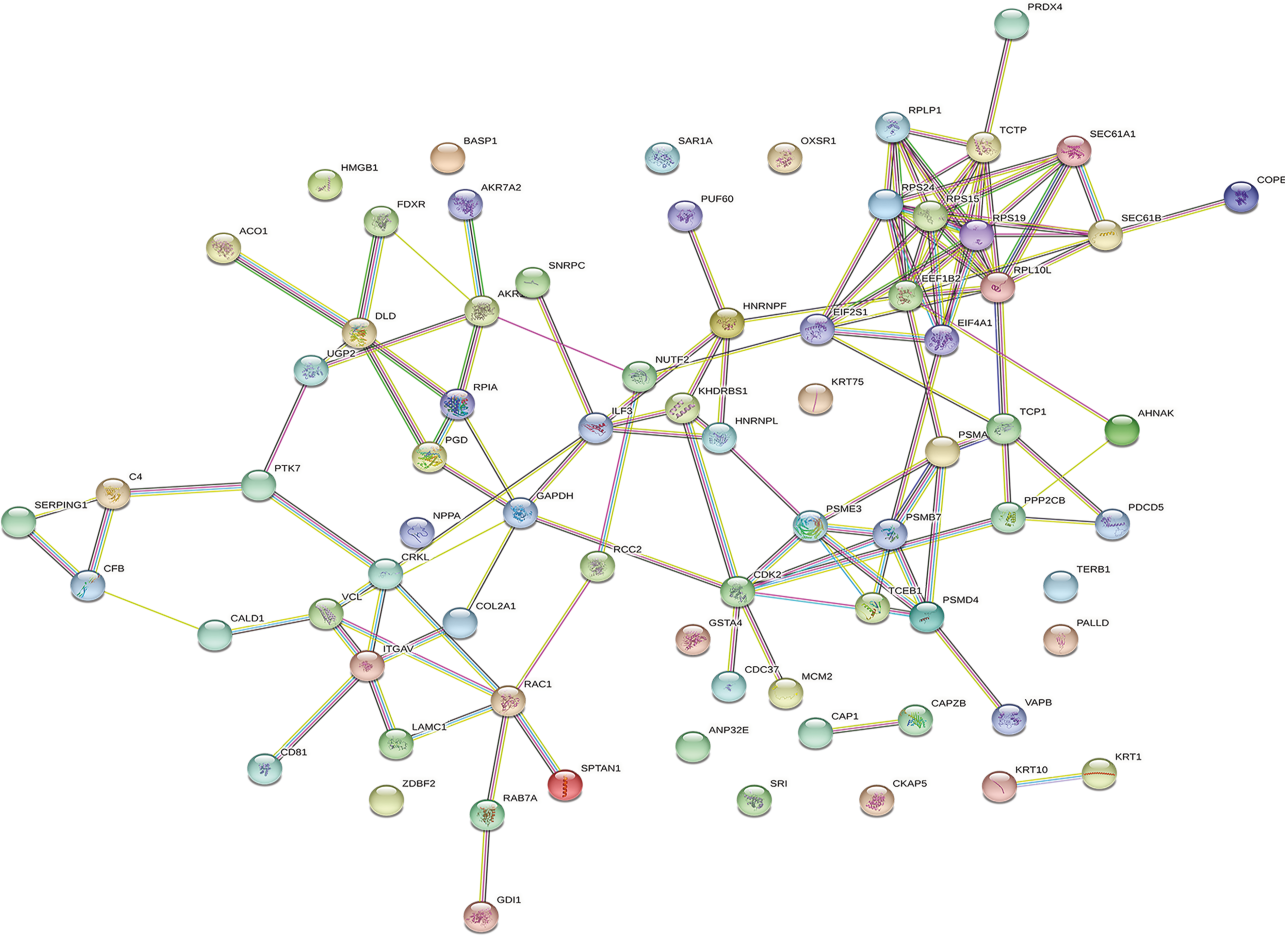
Web network of ovarian proteins altered by ZEN in thermal neutral pre-pubertal gilts. Protein-protein associations of 84 altered ovarian proteins are depicted as a web network. Network nodes represent proteins, with colored nodes indicating the first shell of interactors and white nodes indicating the second shell of interactors. Empty nodes illustrate proteins with unknown 3D structures and filled nodes represent a known or predicted 3D structure. Edges depict protein-protein associations between nodes and illustrate proteins with a shared function. Light blue edges = known interactions curated from databases, light pink edges = experimentally determined known interactions, green edges = gene neighborhood predicted interactions, orange edges = predicted interactions with gene fusions, and navy edges = predicted interactions with gene co-occurrence.

For molecular and cellular pathways, 11 functional classifications were determined, with the top five processes altered by ZEN relative to vehicle control in TN gilts being associated with sec61 translocon complex (40%), rough endoplasmic reticulum membrane (8.2%), proteasome complex (5.4%), myofibril (2.6%), and structural molecule activity (1.9%; FDR ≤ 0.05; Figure 2; Supplementary Table 4). Additionally, the STRING protein-protein interaction network identified 75 nodes and 122 edges associated with protein function to be altered by ZEN in TN gilts (Figure 3).

**Figure 4.**
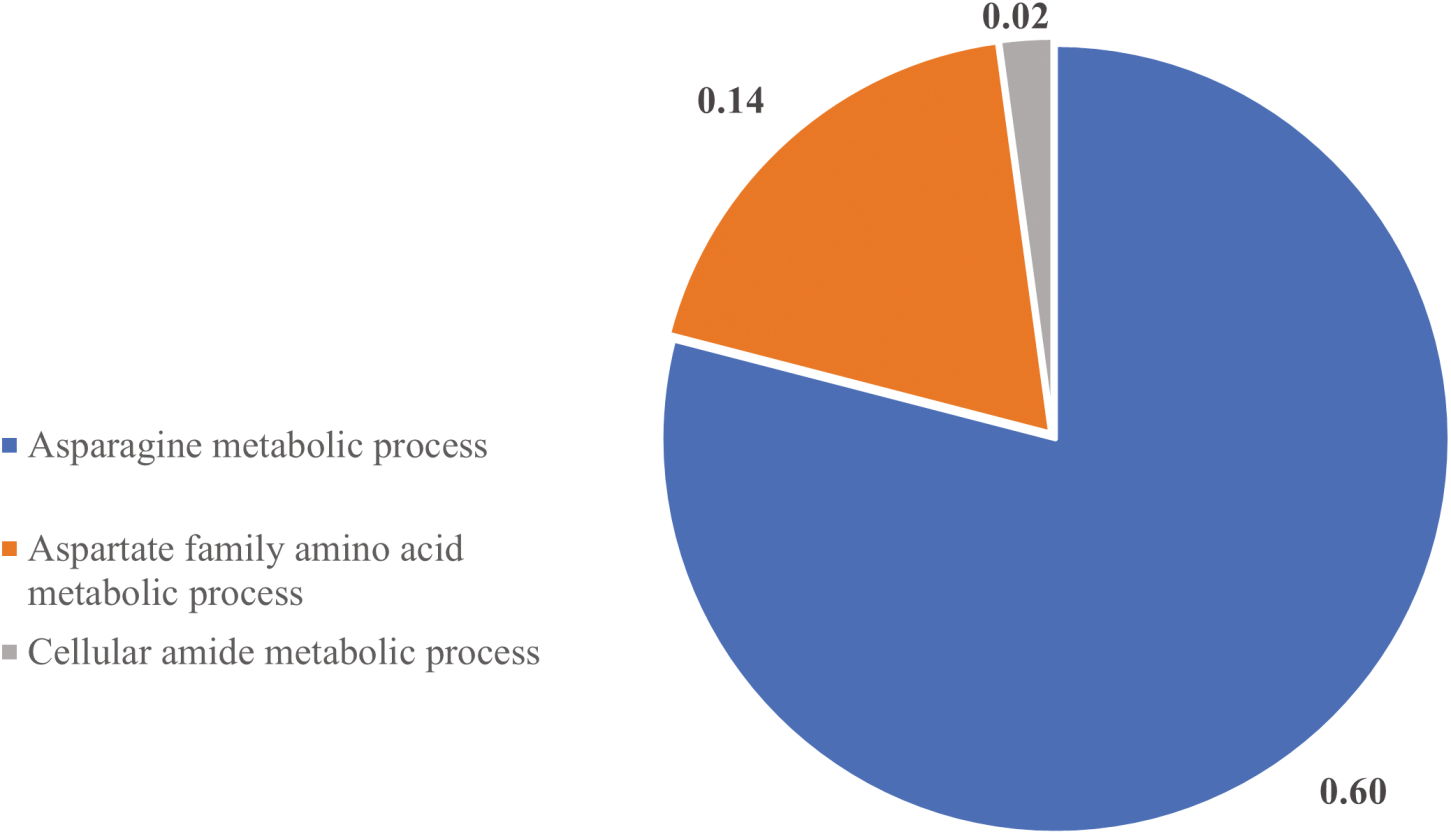
Biological pathway classification of ovarian proteins altered by ZEN exposure in pair-fed females. Pie chart represents the distribution of altered proteins identified in the PC vs. PZ comparison. Biological processes are presented as a percentage, FDR ≤ 0.05.

**Figure 5.**
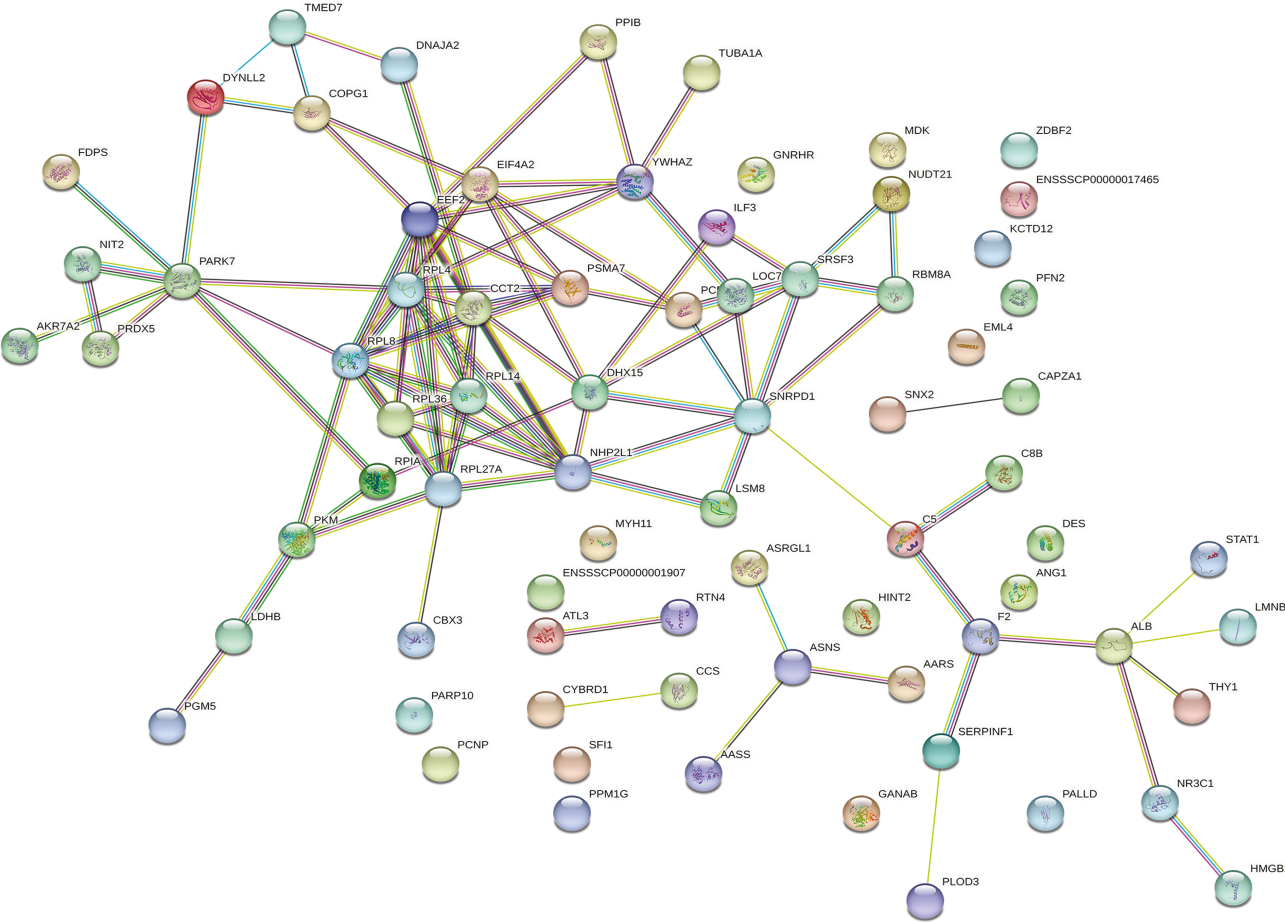
Protein-protein associations ovarian proteins altered by ZEN exposure in pair-fed gilts. Network nodes represent proteins, with colored nodes indicating the first shell of interactors and white nodes indicating second shell of interactors. Empty nodes illustrate proteins with unknown 3D structures and filled nodes represent a known or predicted 3D structure. Edges depict protein-protein associations between nodes and illustrate proteins with a shared function. Light blue edges = known interactions curated from databases, light pink edges = experimentally determined known interactions, green edges = gene neighborhood predicted interactions, orange edges = predicted interactions with gene fusions, and navy edges = predicted interactions with gene co-occurrence. P ≤ 0.05.

STRING identified three biological pathways altered by ZEN in PF gilts, with pathways associated with asparagine metabolic process (0.60 %), aspartate family amino acid metabolic process (0.14%), cellular amide metabolic process (0.02%; FDR ≤ 0.05; Figure 4; Supplementary Table 5). Protein-protein interaction network distinguished 75 nodes and 102 edges to be altered by ZEN in PF gilts (Figure 5).

**Figure 6.**
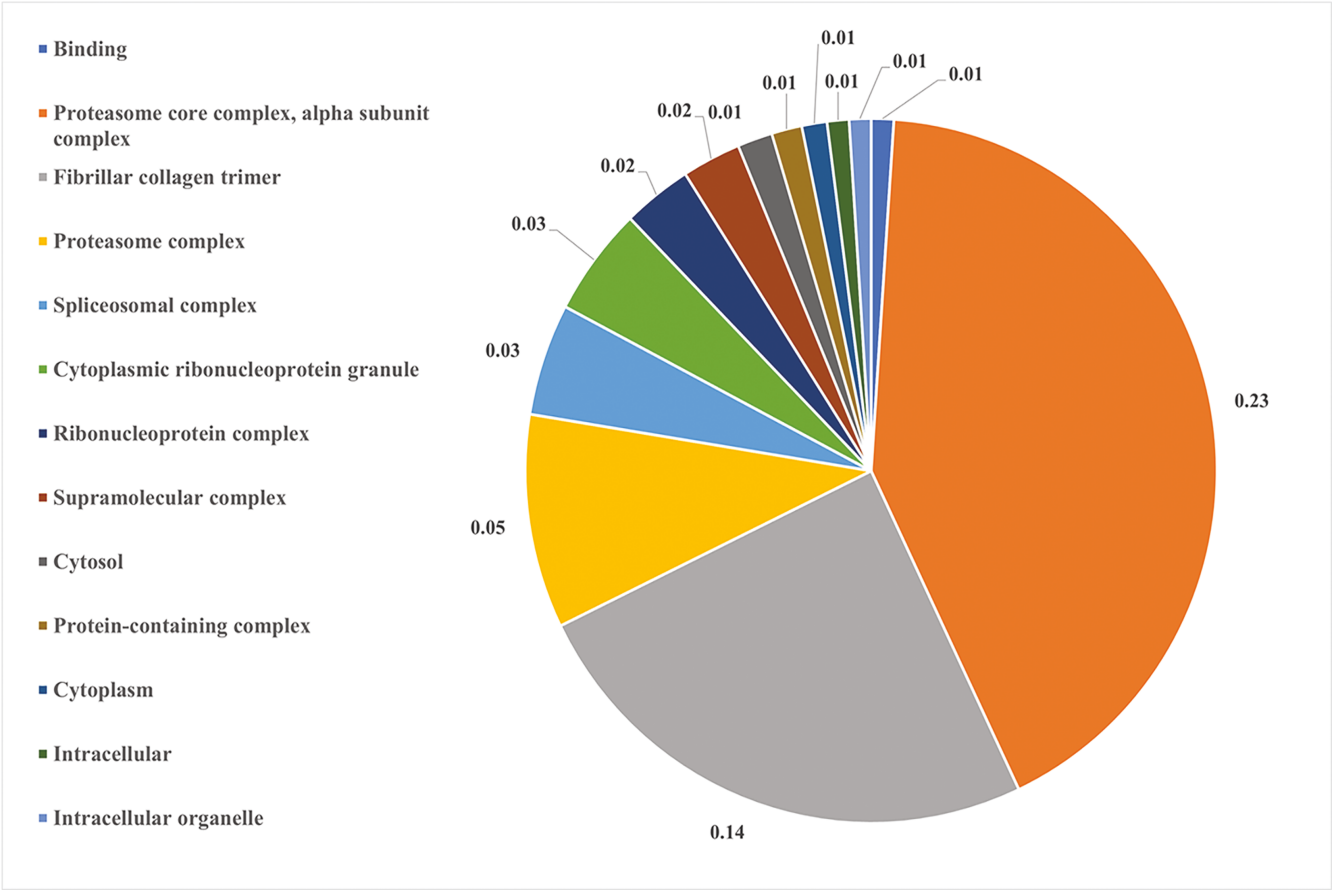
Molecular and cellular pathway classification of ovarian proteins altered by ZEN exposure in heat stress females. Pie chart represents the distribution of altered proteins identified in the HC vs. HZ comparison. Biological processes are presented as a percentage, FDR ≤ 0.05.

**Figure 7.**
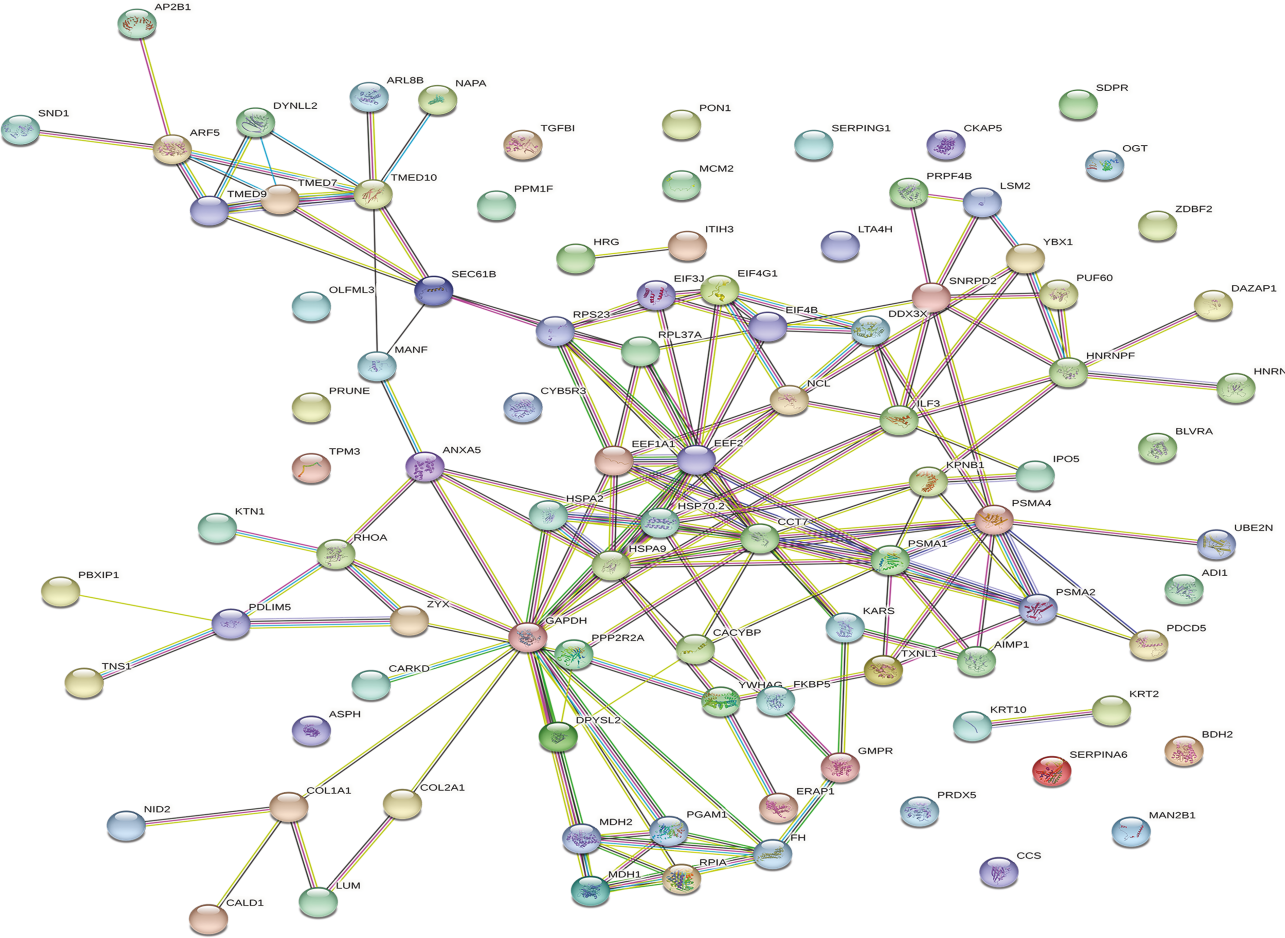
Protein-protein interactions of altered ovarian proteins in heat stress gilts are depicted as a web network. Network nodes represent proteins, with colored nodes indicating the first shell of interactors and white nodes indicating second shell of interactors. Empty nodes illustrate proteins with unknown 3D structures and filled nodes represent a known or predicted 3D structure. Edges depict protein-protein associations between nodes and illustrate proteins with a shared function. Light blue edges = known interactions curated from databases, light pink edges = experimentally determined known interactions, green edges = gene neighborhood predicted interactions, orange edges = predicted interactions with gene fusions, and navy edges = predicted interactions with gene co-occurrence. P ≤ 0.05.

STRING identified 13 molecular and cellular pathways to be altered by ZEN in HS gilts, with the top five pathways associated with proteasome core complex alpha subunit complex (0.23%), fibrillar collagen trimer (0.14%), proteasome complex (0.05%), spliceosomal complex (0.03%), cytoplasmic ribonucleoprotein granule (0.03%; FDR ≤ 0.05; Figure 6; Supplementary Table 6). Protein-protein interaction network identified 95 nodes and 161 edges to be altered by ZEN in HS gilts (Figure 7).

## Identification of Common Proteins and Functions Altered by ZEN Relative to TN/PF and HS Gilts

**Figure 8.**
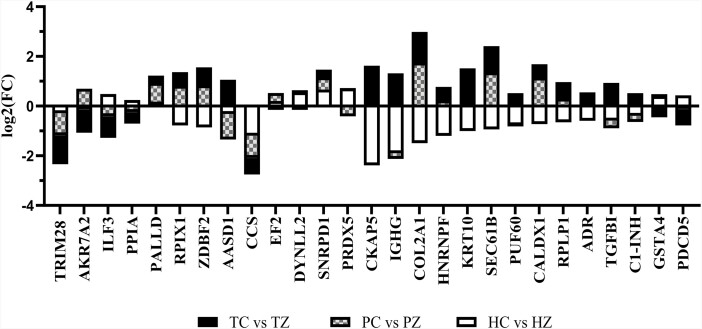
Abundance ovarian proteins altered by ZEN across thermal treatments. Bar chart represents the fold-change of ovarian proteins altered by ZEN in thermal neutral (solid), pair-fed (checkered pattern), or heat stress (open) pre-pubertal gilts. TC: n = 6; TZ: n = 6; PC: n = 6; PZ: n = 6; HC: n = 7; HZ: n = 7; P ≤ 0.05.

**Figure 9.**
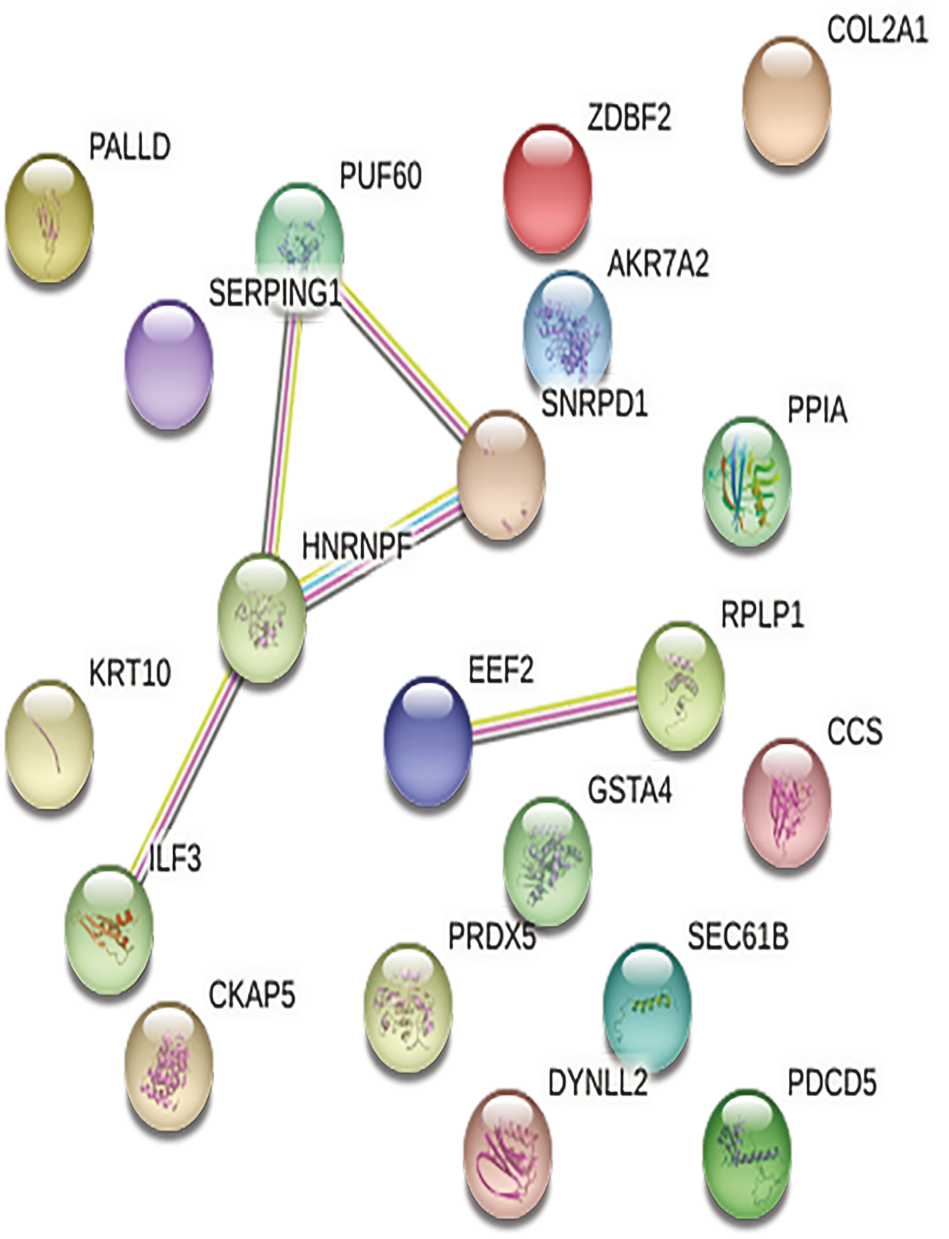
Protein-protein associations of ovarian proteins altered by ZEN independent of thermal treatment depicted as a web network. Network nodes represent proteins, with colored nodes indicating first shell of interactors and white nodes indicating second shell of interactors. Empty nodes illustrate proteins with unknown 3D structures and filled nodes represent a known or predicted 3D structure. Edges depict protein-protein associations between nodes and illustrate proteins with a shared function. Light blue edges = known interactions curated from databases, light pink edges = experimentally determined known interactions, green edges = gene neighborhood predicted interactions, orange edges = predicted interactions with gene fusions, and navy edges = predicted interactions with gene co-occurrence. P ≤ 0.05.

**Figure 10.**
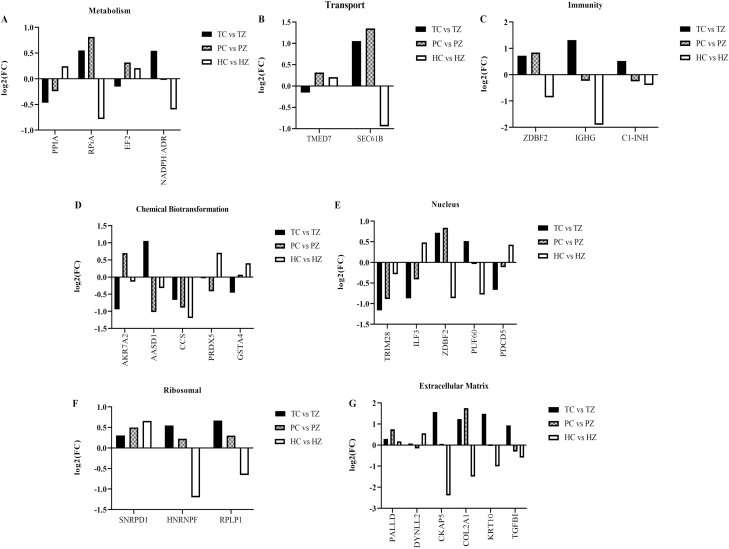
Physiological functions of ovarian proteins are altered by ZEN regardless of thermal treatment. Bar charts represent the fold-change of ovarian proteins altered by ZEN in thermal neutral (solid), PF (checkered pattern), and heat stress (open) pre-pubertal gilts. Proteins are grouped according to the role established by STRING GO analysis: (A) metabolism, (B) transport, (C) immunity, (E) chemical biotransformation, (E) nucleus, (F) ribosomal, and (G) extracellular matrix. TC: n = 6; TZ: n = 6; PC: n = 6; PZ: n = 6; HC: n = 7; HZ: n = 7; P ≤ 0.05.

As previously mentioned, 84, 84, and 108 ovarian proteins were differentially altered by ZEN in TN, PF, and HS gilts, respectively. Of these proteins, 27 ovarian proteins were altered by ZEN regardless of thermal environment, albeit not always in the same direction (Figure 8). Functional protein-protein interactions of common proteins (P ≤ 0.05; Figure 9) depict 20 nodes and 5 edges. No biological processes were identified by STRING. Common proteins are grouped according to functional roles determined by STRING GO analysis (Figure 10).

## Discussion

Recent climate conditions have resulted in intense heat waves which can deleteriously impact human and animal health. Widespread ZEN exposure is also driven by warm and moist climates since improper grain drying can increase ZEN contamination (Bennett and Klich, 2003). Pig reproductive consequences of ZEN consumption are well-documented (Alm et al., 2006; Malekinejad et al., 2007; Jiang et al., 2011; Wan et al., 2022) and this study aimed to identify the ovarian molecular changes stemming from ZEN exposure during TN and HS conditions in the pre-pubertal female.

The ZEN dose utilized is the NOEL level observed in pre-pubertal gilts and is considered low risk for human intake (Joint FAO/WHO Expert Committee on Food Additives, 2000). Feeding a low dose was important to identify ovarian molecular causative changes caused by ZEN exposure without overt ovarian toxicity, since a differential impact of HS was also under investigation. The ZEN-exposed gilts experienced either TN or HS temperatures to mimic the summer months of the Midwestern United States, a period in which pigs endure seasonal infertility. A conserved species response of HS is reduced FI; therefore, a group of TN gilts was included to control for the difference in nutritional intake (the PF group). Pigs undergoing the HS conditions had elevated rectal temperature and respiration rate confirming that HS was successfully induced (Roach et al., 2024). HS decreased FI by 36%, body weight by 3.6%, and decreased average daily gain, with absence of a ZEN effect on FI or body weight (Roach et al., 2024). The age of pigs was purposely chosen to ensure that the observed effects by ZEN in TN and HS pigs were not due to the endogenous hormone milieu.

The mechanisms responsible for ovarian toxicity are diverse and chemical exposures (single or multiple) can impact ovarian function in numerous ways. Thus, to evaluate the consequences of ZEN exposure in the pre-pubertal pig ovary, whole ovary proteomic profiling using LC-MS/MS was employed to measure the altered abundance in proteins between TC vs. TZ, PC vs. PZ, and HC vs. HZ in an unbiased manner, with network analysis providing an understanding of how affected proteins interact in the context of each thermal group. This approach permitted the identification of proteins that were molecular targets of ZEN within thermal group as well as those that were commonly affected by ZEN exposure regardless of thermal environment.

In TN gilts, 84 ovarian proteins were altered by exposure to ZEN, identifying potential targets of ZEN-induced toxicity. Proteins with ovarian roles that were altered by ZEN exposure included PRDX4, AKR1B1, and CDK2. In the ovary, PRDX4 is secreted by cumulus cells and protects the oocyte against oxidative stress (Dai et al., 2022; Qian et al., 2022). There are reports of an association between PRDX4 and both polycystic ovary syndrome (PCOS; Meng et al., 2013; Gateva et al., 2019; Zhou et al., 2021) and ovarian aging (Qian et al., 2016). Exposure to ZEN increased AKR1B1 which is altered during the estrous cycle in the oviduct (Lopera-Vásquez et al., 2022) and functions in control of luteolysis (Chang et al., 2017). This is interesting since these pigs were pre-pubertal in their developmental status. The cell cycle protein CDK2 is altered in the ovary in response to a variety of stressors (Wang et al., 2023a) including xenobiotic exposure (Rhon-Calderón et al., 2018). Additionally, in donkey granulosa cells exposed in culture to ZEN, CDK2 mRNA and protein levels were decreased (Zhang et al., 2018a). In this study, CDK2 was also decreased by ZEN exposure demonstrating consistency across species albeit in different experimental paradigms. Thus, in TN gilts, proteins with roles in luteolysis, oxidative stress, and ovarian pathology were targets of ZEN.

An additional group of PF gilts were included to address the confounding issue of feed reduction due to HS. These gilts were also in TN conditions and whilst these findings could have been included with the TN group, understanding the proteomic effects of ZEN exposure in the PF gilts could also be useful to situations in which underfeeding is also present. Proteins amongst the 84 proteins that were altered by ZEN in PF gilts that have interesting ovarian roles were YWHAZ and STAT1. Exposure to ZEN increased YWHAX in the ovary and YWHAZ has a function in conferring luteal sensitivity to prostaglandin F2α (Goravanahally et al., 2009) and is also suggested to be involved in the transition from the follicular to the luteal phase of the estrous cycle (Zhang et al., 2019). Increased by exposure to TNF-α in cumulus cells in the bovine ovary (Piersanti et al., 2019), STAT1 was decreased by ZEN exposure in the PF gilt ovary. Interestingly there is a relationship between the metabolic hormone, ghrelin, and STAT1 in granulosa cells of the ovary (Benco et al., 2009), and since the gilts in the study are limited fed to match the HS FI level, could reflect an interaction between nutritional status and ZEN exposure.

In the HS gilts, a greater number of proteins in totality were altered by ZEN exposure (108) and included proteins with documented ovarian function, namely DAZAP1, FKBP5, DDX3X, HSPA9, HSPA1A, HSPA2, TXNL1, MGST1, and PPP2R2A. Exposure to ZEN increased DAZAP1 which functions in male germ cells (Vera et al., 2002) and is present in luteal cells of human and rat ovaries (Pan et al., 2005). *Dazap1*^−/−^ mice are infertile with reduced ovarian size (Hsu et al., 2008). Another protein with luteal function, TNXL1 (Pokharel et al., 2020) was decreased by ZEN exposure in HS gilt ovaries. Ovarian FKBP5 was increased by ZEN in HS gilts. In fetal ovaries, exposure to dexamethasone increased FKBP5 (Poulain et al., 2012), and overexpression of FKBP5 was associated with chemoresistance in ovarian cancer cells (Sun et al., 2014), suggesting that ovarian FKBP5 is involved in the ovarian response to xenobiotic exposure. DDX3X was increased by ZEN in HS gilt ovaries and is involved with cell survival and cell cycle control in embryonic development (Li et al., 2014) and is also identified as being a potential regulator of ovulation (Zaniker et al., 2023). As an indicator of ZEN-induced oxidative stress in gilt ovaries during HS, MGST1 is increased in oocytes from endometriosis patients (Ferrero et al., 2019), and is increased by antioxidant treatment of PCOS in rats (Zhou et al., 2021). In this study, MGST1 was decreased by ZEN exposure and could reflect that the protein was depleted through functioning in the response to oxidative stress or could also be attributable to a mode of toxicity of ZEN exposure.

Three HSPs were decreased in response to ZEN exposure in HS gilt ovaries: HSPA1A, HSPA2, and HSPA9, perhaps unsurprising considering their roles in the ovarian response to HS (Abdelnour et al., 2020) and to ZEN exposure (Chen et al., 2019; Yi et al., 2022). In cycling pig ovaries, HSPA1A is responsive to HS or lipopolysaccharide exposure alone in the absence of ZEN exposure (Seibert et al., 2019). Supplementation of oocytes with the antioxidant melatonin, reduced *HSPA1A* mRNA in resultant bovine blastocysts (Cordova et al., 2022). A role for HSPA2 in primordial to primary follicle transition is also supported in pig ovaries (Xu et al., 2017) and cultured granulosa cells increased *Hspa2* mRNA abundance in response to nitropropionic acid as an indicator of oxidative stress induction (Kang et al., 2018) Thus, HSPs are increased in response to ZEN and HS, are markers of oxidative stress, and this study indicates that combination of both alters the abundance of three HSP in response to ZEN exposure in HS gilt ovaries. Finally, PPP2R2A is involved in ovarian cancer biology (Youn and Simon, 2013; Zhang et al., 2018b) and ZEN exposure promotes tumorigenesis in granulosa cells (Zhang et al., 2018c). PPP2R2A was decreased to the greatest extent of all proteins affected by ZEN in the HS gilts. Thus, in combination with HS, while a greater number of proteins were altered in abundance by ZEN exposure, similar ovarian roles in the oxidative stress response, apoptosis, and luteolysis were noted.

As an effort to identify proteins that were commonly affected by ZEN exposure independent of thermal load, a comparison of the altered proteins across treatments was made. Proteins with functional roles associated with metabolism (PPIA, RPIA, EF2, and ADR), immune response (ZDBF2, IGHG, and C1-INH), detoxification (AKR7A2, AASD1, CCS, PRDX5, and GSTA4) and transport (TMED7 and SEC61B) were identified to be altered by ZEN regardless of thermal exposure, albeit sometimes in opposing directions of change. Additionally, cellular locations where those changes occurred were nuclear, ribosomal, and in the extracellular matrix.

Four proteins were altered by ZEN exposure in the same pattern of change across thermal groups: TRIM28 (decreased), PALLD (increased), CCS (decreased), and SNRPD1 (increased). TRIM28 is decreased by ZEN exposure in TN and HS gilt ovaries. Interestingly, loss of TRIM28 resulted in differentiation of ovarian granulosa cells to Sertoli cells which was dependent upon sumoylation (Rossitto et al., 2022) suggesting that a ZEN-induced reduction would detrimentally affect ovarian function. ZEN exposure increased PALLD in all three thermal groups. While ovarian roles for PALLD are not widely known, increased PALLD is associated with ovarian high-grade serous carcinoma (Davidson et al., 2020), potentially a contraindication of ZEN exposure. Accumulation of radical oxygen species is lessened by the action of CCS (Culotta et al., 1997) and CCS was decreased by ZEN exposure in all thermal groups potentially lessening the ovarian capacity to respond to oxidative stress. An oncogene, SNRPD1, lacks clearly defined ovarian roles (Liu et al., 2022; Dai et al., 2023; Wang et al., 2023b) although small nuclear ribonucleoproteins have been visualized in the germinal vesicle stage oocytes, potentially indicating a role for RNRPD1 in oocyte function. Thus, the four proteins altered in the same pattern of change by ZEN exposure have physiological roles that if disrupted by ZEN exposure could be detrimental to ovarian function.

Of the other proteins altered by ZEN in all thermal groups but not necessarily in the same pattern of change, those with known ovarian roles are TGFB1 which participates in primordial follicle activation (Pangas, 2012; Chen et al., 2022), luteal regression (Monaco and Davis, 2023) and regulation of extracellular matrix genes (Harlow et al., 2002). Though there are fewer defined roles for ovarian PDCD5, there are links with ovarian pathology including ovarian cancer (Zhang et al., 2011) and granulosa cell apoptosis (Geng et al., 2022).

Relevant to detoxification and oxidative stress, GSTA4 is a phase II detoxifying enzyme that metabolizes electrophiles and carcinogens (Hubatsch et al., 1998) and it is decreased due to ZEN exposure in TN but increased in HS pig ovaries. The AKR7A2 protein detoxifies aldehydes and ketones and catalyzes the reduction of xenobiotics (Barski et al., 2008) and is increased by ZEN exposure in both TN groups but decreased in HS gilt ovaries. Similar to PRDX4 which was altered in TN gilts exposed to ZEN, PRDX5 responds to oxidative stress and induces proinflammatory cytokines in macrophages through activation of toll-like receptor 4 (Poncin et al., 2021). Both TN and PF had reduced levels of PRDX5 while HS gilts had higher PRDX5 in response to ZEN. The granulosa cell transcriptome from primordial follicles had higher *Prdx5* relative to primary follicles (Ernst and Lykke-Hartmann, 2018). Thus, ZEN exposure during HS increased the abundance of GST4A and PRDX5 suggesting an effort by the ovary to relieve oxidative and xenobiotic-induced stress.

In conclusion, this study identifies targets of ZEN as modes of toxicity. In addition, different thermal load paradigms resulted in differential ovarian responses to ZEN. The ovarian abundance of proteins with documented ovarian roles and those that function in metabolism, immune response, detoxification, and transport were identified as targets of ZEN. Taken together, the data identify ZEN-induced ovarian alterations and support that the ovarian response to ZEN is different in TN relative to HS pigs, suggesting that hyperthermia can impact the outcome of ovarian xenobiotic exposure. Additionally, ovarian proteins consistently affected by ZEN exposure across thermal treatments could indicate potential mitigation targets to mitigate ZEN-induced reproductive toxicity.

## Supplementary Material

skae115_suppl_Supplementary_Tables_1

skae115_suppl_Supplementary_Tables_2

skae115_suppl_Supplementary_Tables_3

## Abbreviations

α-ZOL: alpha-zearalenone
β-ZOL: beta-zearalenone
BCA: bicinchoninic acid
CT: control
E_2_: 17β-estradiol
FDR: false discovery rate;
FI: feed intake
GO: gene ontology
HC: heat stress control
HS: heat stress
HZ: heat stress zearalenone
KEGG: Kyoto encyclopedia of genes and genomes
LC: liquid chromatography
LC-MS/MS: liquid chromatography-tandem mass spectrometry
NOEL: no observed effect level
PC: pair-fed vehicle control
PI3K: phosphatidylinositol-3 kinase
PRTC: peptide retention time calibration
PF: pair-fed
PZ: pair-fed zearalenone
STRING: search tool for the retrieval of interacting genes/proteins
TC: thermoneutral vehicle control
TN: thermoneutral
TZ: thermoneutral zearalenone
ZEN: zearalenone
