# Supplementary material for: Zearalenone exposure differentially affects the ovarian proteome in pre-pubertal gilts during thermal neutral and heat stress conditions
Source: J Anim Sci. 2024 Apr 26;102:skae115. doi: 10.1093/jas/skae115 (PMC11217906; doi:10.1093/jas/skae115)
Supplement: skae115_suppl_Supplementary_Tables_1 [file skae115_suppl_supplementary_tables_1.docx]

| **Uniprot ID** | **Protein name** | **Protein abbreviation** | **log2(FC)** |
| --- | --- | --- | --- |
| F1SGI7 | Keratin 75 | KRT75 | 2.288 |
| A1Z1R6 | Integrin alpha V | ITGAV | 1.966 |
| F1SQ01 | Peroxiredoxin | PRDX4 | 1.699 |
| A0A4X1VSE7 | Cytoskeleton associated protein 5 | CKAP5 | 1.563 |
| A0A0B8S0B1 | Adenylyl cyclase-associated protein | CAP1 | 1.503 |
| F1SGG3 | Keratin 1 | KRT1 | 1.498 |
| I3LDS3 | Keratin 10 | KRT10 | 1.482 |
| A0A4X1V580 | Tyrosine kinase 7 | PTK7 | 1.445 |
| L8B0U8 | IgG heavy chain | IGHG | 1.314 |
| A0A287A2Q7 | Collagen type II alpha 1 chain | COL2A1 | 1.233 |
| A0A287AAP0 | Fumarylacetoacetate hydrolase domain-containing protein 2A isoform X3 | FAHD2A3 | 1.131 |
| A0A4X1SH59 | SEC61 translocon alpha 1 subunit beta | SEC61B | 1.058 |
| A0A481ARR7 | Alpha-aminoadipic semialdehyde dehydrogenase | AASA | 1.056 |
| A0A4X1TV07 | SEC61 translocon alpha 1 subunit alpha 1 | SEC61A1 | 1.025 |
| A0A480X877 | Transforming growth factor-beta-induced protein | TGFBI | 0.930 |
| F1RKX9 | CRK like proto-oncogene, adaptor protein | CRKL | 0.883 |
| F1S663 | Laminin subunit gamma 1 | LAMC1 | 0.795 |
| A0A480T5S7 | Ras-related protein Rab-7a isoform X1 | RAB7A | 0.757 |
| A0A4X1US63 | Vesicle-associated membrane protein-associated protein B | VAPB | 0.735 |
| A0A4X1V152 | Zinc finger DBF-type containing 2 | ZDBF2 | 0.717 |
| A0A4X1T568 | Ribosomal protein lateral stalk subunit P1 | RPLP1 | 0.667 |
| P09623 | Dihydrolipoyl dehydrogenase | DLD | 0.657 |
| A0A480LQF4 | Cluster of differentiation 81 | CD81 | 0.625 |
| A0A480W9J0 | Regulator of chromosome condensation 2 | RCC2 | 0.615 |
| F1SHD6 | Eukaryotic translation elongation factor 1 beta 2 | EEF1B2 | 0.577 |
| A0A481BC81 | Ribose-5-phosphate isomerase | RPIA | 0.551 |
| A0A4X1VKL9 | Desmoyokin | AHNAK | 0.550 |
| F1RG16 | Heterogeneous nuclear ribonucleoprotein F | HNRNPF | 0.547 |
| A0A4X1SGU8 | Ferredoxin reductase | FDXR | 0.544 |
| A0A480TAI8 | Caldesmon isoform X1 | CALD1 | 0.543 |
| A0A4X1U2A6 | UvrD-like helicase | URVD | 0.524 |
| A0A4X1UVY8 | 60 kDa Poly(U)-binding-splicing-factor | PUF60 | 0.518 |
| A0A480W9F9 | Plasma protease C1 inhibitor | SERPING1 | 0.517 |
| A0A4X1UMC0 | Aconitase 1 | ACO1 | 0.507 |
| P80276 | Aldo-keto reductase family 1 member B1 | AKR1B1 | 0.402 |
| A0A4X1U6V4 | Canopy FGF signaling regulator 4 | CNPY4 | 0.374 |
| A0A4X1U4D4 | Eukaryotic translation initiation factor 2 subunit 1 | EIF2S1 | 0.367 |
| A0A4X1SRF3 | Vinculin | VCL | 0.333 |
| A0A287AR67 | Sorcin | SRI | 0.302 |
| A0A480HYR0 | Spectrin alpha chain, non-erythrocytic | SPTAN1 | 0.299 |
| F1RIW3 | Palladin | PALLD | 0.291 |
| A0A480J4N2 | Complement C4-A | C4A | 0.265 |
| A0A4X1TM42 | Brain abundant membrane attachment signal protein 1 | BASP1 | 0.265 |
| A0A286ZI08 | UTP--glucose-1-phosphate uridylyltransferase | UGP2 | 0.221 |
| A0A4X1TPR9 | T-complex protein 1 | TCP1 | 0.115 |
| A0A480YSA5 | 6-phosphogluconate dehydrogenase | PGD | -0.260 |
| A9XFX6 | F-actin-capping protein subunit beta | CAPZB | -0.289 |
| I3L893 | Rab GDP dissociation inhibitor | GDI1 | -0.308 |
| A0A4X1SDD8 | 40s ribosomal protein S19 | RPS19 | -0.310 |
| K7GPT9 | Complement factor B | CFB | -0.356 |
| A0A4X1TY74 | Vicinal oxygen chelate domain-containing protein | VOC | -0.358 |
| A0A480F5K0 | Heterogeneous nuclear ribonucleoprotein L | HNRNPL | -0.377 |
| P00355 | Glyceraldehyde-3-phosphate dehydrogenase | GAPDH | -0.382 |
| A0A4X1TJK2 | High mobility group box 1 | HMGB1 | -0.410 |
| A0A287ARC7 | Cyclin dependent kinase 2 | CDK2 | -0.431 |
| A0A480ISP4 | 26S protease regulatory subunit 4 | PSMD4 | -0.434 |
| A0A4X1TD81 | Glutathione S-transferase alpha 4 | GSTA4 | -0.458 |
| A0A4X1U6K8 | Coatomer subunit epsilon | COPE | -0.484 |
| I3LP11 | KH RNA binding domain containing, signal transduction associated 1 | KHDRBS1 | -0.536 |
| A0A096ZXA2 | Oxidative-stress responsive kinase 1 | OXSR1 | -0.558 |
| A0A4X1VTD3 | Protein phosphatase 2 catalytic subunit beta | PPP2CB | -0.566 |
| Q684M6 | Cell division cycle 37 | CDC37 | -0.583 |
| A0A4X1TPV7 | Rac family small GTPase 1 | RAC1 | -0.585 |
| A0A480SCD0 | Proteasome subunit alpha type | PSMA5 | -0.610 |
| A0A480NR20 | Ubiquitin carboxyl-terminal hydrolase | UCH | -0.628 |
| A0A481D4P9 | Programmed cell death protein 5 | PDCD5 | -0.666 |
| A0A4X1TYN7 | Acidic leucine-rich nuclear phosphoprotein 32 family member | ANP32E | -0.697 |
| A0A480Y209 | Minichromosome maintenance complex component 7 | MCM7 | -0.787 |
| A0A4X1T4T3 | Small nuclear ribonucleoprotein polypeptide C | SNRPC | -0.866 |
| K7GL83 | Interleukin enhancer binding factor 3 | ILF3 | -0.872 |
| A0A4X1UHL0 | Nuclear transport factor 2 | NUTF2 | -0.912 |
| A0A5G2R0M9 | Aldo-keto reductase family 7 | AKR7A2 | -0.945 |
| Q52NJ3 | Secretion associated Ras related GTPase 1A | SAR1A | -1.016 |
| A0A287A8V1 | Eukaryotic translation initiation factor 4A1 | EIF4A1 | -1.105 |
| A0A5G2QXY8 | Elongin C | ELOC | -1.157 |
| A0A4X1W3I5 | Tripartite motif containing 28 | TRIM28 | -1.163 |
| A0A4X1W9F5 | Ubiquitin conjugating enzyme E2 M | UBE2M | -1.170 |
| A1XQU1 | Proteasome subunit beta type-7 | PSMB7 | -1.294 |
| A0A4X1T5U8 | 60s ribosomal protein L10 | RPL10 | -1.406 |
| I3LJP6 | 40S ribosomal protein S24 | RPS24 | -1.422 |
| A0A4X1TQS8 | CDP-diacylglycerol synthase | CDP | -1.711 |
| A0A4X1VTH3 | 40S ribosomal protein S15 | RPS15 | -1.891 |
| A0A4X1TXU2 | Proteasome activator complex subunit 3 | PSME3 | -1.932 |
| A0A287B4E6 | Translationally-controlled tumor protein | TPT1 | -2.405 |

**log2(FC) = Log two-fold change in TZ relative to TC gilt ovaries**
