# Supplementary material for: Zearalenone exposure differentially affects the ovarian proteome in pre-pubertal gilts during thermal neutral and heat stress conditions
Source: J Anim Sci. 2024 Apr 26;102:skae115. doi: 10.1093/jas/skae115 (PMC11217906; doi:10.1093/jas/skae115)
Supplement: skae115_suppl_Supplementary_Tables_2 [file skae115_suppl_supplementary_tables_2.docx]

**Supplementary Table 2. Effect of ZEN exposure on ovarian protein abundance in PF gilts**

| **Uniprot ID** | **Protein name** | **Protein abbreviation** | **log2(FC)** |
| --- | --- | --- | --- |
| F6Q1M2 | Asparagine synthetase | ASNS | 2.200 |
| A0A4X1VRC0 | Midkine | MDK | 2.166 |
| A0A4X1SRP3 | Alanine tRNA ligase | AARS | 1.900 |
| A0A2A2XAK5 | GNAT family N-acetyltransferase | FGO86_01900 | 1.342 |
| Q29582 | Pyruvate kinase, muscle | PKM | 1.294 |
| A0A286ZKB4 | Complement C5 | C5 | 1.162 |
| A0A4X1V4X7 | SFI1 centrin binding protein | SFI1 | 1.062 |
| I3LUD6 | Cytochrome b reductase 1 | CYBRD1 | 0.917 |
| A0A4X1UHX7 | Lamin B1 | LMNB1 | 0.904 |
| F1S4U9 | EMAP like 4 | EML4 | 0.857 |
| A0A4X1V152 | Zinc finger DBF-type containing 2 | ZDBF2 | 0.838 |
| A0A481BC81 | Ribose-5-phosphate isomerase | RPIA | 0.814 |
| P02540 | Desmin | DES | 0.784 |
| A0A287B5Q4 | Reticulon | RTN4 | 0.783 |
| A0A480X352 | Nucleoside diphosphate kinase | NDPK | 0.753 |
| F1RIW3 | Palladin isoform X1 | PALLD | 0.752 |
| A0A480KD21 | Lysyl hydroxylase | LH | 0.751 |
| A0A287BBY5 | DEAH-box helicase 15 | DHX15 | 0.744 |
| A0A5G2R0M9 | Aldo-keto reductase family 7 | AKR7A2 | 0.700 |
| A0A4X1VJU1 | Atlastin GTPase 3 | ATL3 | 0.699 |
| A0A5G2QJ53 | Nudix Hydrolase 21 | NUDT21 | 0.671 |
| F2Z5P9 | LSM8 homolog, U6 small nuclear RNA associated | LSM8 | 0.617 |
| A0A4X1UI30 | Procollagen-lysine, 2-oxoglutarate 5-dioxygenase 3 | PLOD3 | 0.607 |
| A0A2I6SB80 | Thy-1 cell surface antigen | THY1 | 0.572 |
| A0A4X1UBF3 | Eukaryotic translation initiation factor 4A2 | EIF4A2 | 0.546 |
| A0A287APE8 | ITPR interacting domain containing 2 | ITPRID2 | 0.518 |
| A0A4X1UXB1 | PEST proteolytic signal-containing nuclear protein | PCNP | 0.516 |
| A2THZ2 | Albumin | ALB | 0.505 |
| A0A4X1VMY1 | Small nuclear ribonucleoprotein D1 polypeptide | SNRPD1 | 0.499 |
| A0A4X1SY39 | Profilin 2 | PFN2 | 0.492 |
| K9IVW4 | Myotrophin | MTPN | 0.461 |
| P00336 | L-lactate dehydrogenase B chain | LDHB | 0.447 |
| A0A480HYZ5 | Myosin heavy chain 11 | MYH11 | 0.441 |
| F1SKY2 | Nitrilase family member 2 | NIT2 | 0.404 |
| F1RJ01 | High mobility group protein box 2 | HMGB2 | 0.381 |
| Q2EHH8 | Protein phosphatase 1 catalytic subunit alpha | PPP1CA | 0.340 |
| A0A5G2R2A7 | 60S ribosomal protein L4 | RPL4 | 0.337 |
| A0A287AWI9 | Elongation factor 2 | EEF2 | 0.317 |
| A0A287AWS4 | 60s ribosomal protein L27a | RPL27A | 0.316 |
| A0A480LRK1 | Ribonuclease inhibitor 1 | RNH1 | 0.312 |
| D0G0C8 | Chaperonin containing TCP1 subunit 2 | CCT2 | 0.309 |
| A0A480M4R9 | COPI coat complex subunit gamma 1 | COPG1 | 0.278 |
| A0A287BHL0 | Poly(rC) binding protein 2 | PCBP2 | 0.244 |
| Q0R678 | Parkinsonism associated deglycase | PARK7 | 0.203 |
| A0A4X1TV95 | Tyrosine 3-monooxygenase/tryptophan 5-monooxygenase activation protein zeta | YWHAZ | 0.178 |
| A0A4X1SKB8 | Dynein light chain | DYNLL2 | -0.156 |
| F1RY92 | Serine and arginine rich splicing factor 3 | SRSF3 | -0.212 |
| A0A4X1U2I6 | Peptidyl-prolyl cis-trans isomerase | PPIB | -0.233 |
| A0A480P4D2 | Complement factor I | CF1 | -0.310 |
| A0PFK5 | Capping actin protein of muscle Z-line subunit alpha 1 | CAPZA1 | -0.313 |
| A0A287B217 | Plectin | PLEC | -0.336 |
| A0A4X1UXP5 | Proteasome subunit alpha type 7 | PSMA7 | -0.396 |
| K7GL83 | Interleukin enhancer binding factor 3 | ILF3 | -0.413 |
| A0A5G2R9D6 | Peroxiredoxin 5 | PRDX5 | -0.420 |
| A0A4X1VTP8 | Coagulation factor II, thrombin | F2 | -0.437 |
| A0A4X1UQ84 | 60s ribosomal protein L8 | RPL8 | -0.445 |
| A1XQU3 | 60s ribosomal protein L14 | RPL14 | -0.458 |
| A0A286ZN85 | Signal transducer and activator of transcription | STAT1 | -0.484 |
| A0A287B5W2 | Trypsinogen | LOC100302368 | -0.494 |
| A0A287AYJ8 | Serpin family F member 1 | SERPINF1 | -0.499 |
| F1RP05 | DnaJ heat shock protein family (Hsp40) member A2 | DNAJA2 | -0.522 |
| I3LI59 | RNA-binding protein 8A | RBM8A | -0.645 |
| A0A4X1VNE4 | Asparaginase and isoaspartyl peptidase 1 | ASRGL1 | -0.754 |
| A0A4X1TPG2 | Protein phosphatase, Mg2+/Mn2+ dependent 1G | PPM1G | -0.850 |
| B1PSB6 | Adiponectin | ADIPOQ | -0.864 |
| A0A4X1VTQ0 | Histidine triad nucleotide binding protein 2 | HINT2 | -0.878 |
| A0A4X1W3I5 | Tripartite motif containing 28 | TRIM28 | -0.889 |
| A0A4X1TJN9 | Copper chaperone for superoxide dismutase | CCS | -0.893 |
| A0A480F699 | Glucosidase II alpha subunit | GANAB | -0.896 |
| A0SEH2 | Complement component C8B | C8B | -0.932 |
| F1SJE6 | Phosphoglucomutase 5 | PGM5 | -0.996 |
| A0A481ARR7 | Alpha-aminoadipic semialdehyde dehydrogenase | AASS | -1.023 |
| A0A5G2QST4 | Farnesyl diphosphate synthase | FDPS | -1.094 |
| A0A480QWI4 | Histone H1.4 | H1-4 | -1.200 |
| A0A287B0T7 | Chromobox protein homolog 3 | CBX3 | -1.230 |
| A0A4X1SLB9 | Superkiller viralicidic activity 2-like 2 | SKIC2 | -1.289 |
| A0A4X1UC57 | Hepatocyte growth factor-regulated tyrosine kinase substrate | HGS | -1.354 |
| A0A287BMK7 | Sorting nexin 2 | SNX2 | -1.383 |
| F1RHE6 | Potassium channel tetramerization domain containing 12 | KCTD12 | -1.545 |
| A0A286ZYJ8 | UPF1 RNA helicase and ATPase | UPF1 | -1.740 |
| A0A4X1VRZ2 | 60s ribosomal protein L36 | RPL36 | -1.860 |
| A0A286ZPD7 | Transmembrane emp24 domain-containing protein 7 | TMED7 | -2.099 |
| P02550 | Tubulin alpha-1A chain | TUBA1A | -2.380 |
| F2Z5V3 | Small nuclear ribonucloprotein 13 | SNU13 | -2.474 |

**log2(FC) = Log two-fold change in PZ relative to PC gilt ovaries**
