# Supplementary material for: Zearalenone exposure differentially affects the ovarian proteome in pre-pubertal gilts during thermal neutral and heat stress conditions
Source: J Anim Sci. 2024 Apr 26;102:skae115. doi: 10.1093/jas/skae115 (PMC11217906; doi:10.1093/jas/skae115)
Supplement: skae115_suppl_Supplementary_Tables_3 [file skae115_suppl_supplementary_tables_3.docx]

**Supplementary Table 3. Impact of ZEN exposure on ovarian protein abundance in HS gilts**

| **Uniprot ID** | **Protein name** | **Protein abbreviation** | **log2(FC)** |
| --- | --- | --- | --- |
| A0A480WWN6 | Eukaryotic translation initiation factor 4B | EIF4B | 1.559 |
| A0SNU7 | Glyceraldehyde-3-phosphate dehydrogenase | GAPDH | 1.506 |
| A0A4X1VTJ5 | DAZ associated protein 1 | DAZAP1 | 1.469 |
| A0A480KGP4 | Inter-alpha-trypsin inhibitor heavy chain 3 | ITIH3 | 1.180 |
| I3LDR9 | Caveolae associated protein 2 | CAVIN2 | 0.990 |
| A0A480Z819 | Eukaryotic translation initiation factor 3 subunit C | EIF3C | 0.989 |
| A0A4X1UIX9 | Ribosomal protein L37a | RPL37A | 0.880 |
| Q06A96 | Small nuclear ribonucleoprotein polypeptide B2 | SNRPB2 | 0.771 |
| A0A286ZQD3 | FKBP prolyl isomerase 5 | FKBP5 | 0.770 |
| A0A4X1UJZ2 | Acireductone dioxygenase 1 | ADI1 | 0.758 |
| A0A4X1UM40 | Tensin-1 | TNS1 | 0.728 |
| A0A5G2R9D6 | Peroxiredoxin | PRDX5 | 0.711 |
| F2Z5W4 | Heterogeneous nuclear ribonucleoprotein H3 | HNRNPH3 | 0.683 |
| A0A4X1TGG9 | Threonyl-tRNA synthetase 1 | TARS1 | 0.667 |
| F2Z512 | 40S ribosomal protein S23 | RPS23 | 0.638 |
| B9V4F0 | ADP-ribosylation factor 5 | ARF5 | 0.624 |
| A0A480Y209 | DNA replication licensing factor MCM7 | MCM7 | 0.600 |
| Q29014 | Orosomucoid 1 | ORM | 0.590 |
| A0A287A1G4 | PDZ and LIM domain 5 | PDLIM5 | 0.586 |
| A0A4X1TT06 | Paraoxonase 1 | PON1 | 0.582 |
| A0A4X1SKB8 | Dynein light chain | DYNLL2 | 0.560 |
| A0A480PTU4 | Lysyl-tRNA synthetase | KARS | 0.549 |
| I3LAB6 | Proteasome 20s subunit alpha 2 | PSMA2 | 0.516 |
| A0A4X1THY9 | Fumarate hydratase | FH | 0.496 |
| A0A4X1WBK5 | Keratin 2 | KRT2 | 0.493 |
| K7GL83 | Interleukin enhancer binding factor 3 | ILF3 | 0.484 |
| A0A480EV23 | Calpain-1 catalytic subunit | CCS | 0.474 |
| A0A4X1UQF4 | Eukaryotic translation initiation factor 4 gamma 1 | EIF4G1 | 0.471 |
| A0A4X1ST20 | Ras homolog family member A | RHOA | 0.443 |
| A0A481D4P9 | Programmed cell death protein 5 | PDCD5 | 0.431 |
| A0A481B9A6 | Histidine-rich glycoprotein | HRG | 0.414 |
| I3LFV4 | Y-box binding protein 1 | YBX1 | 0.411 |
| A0A287AVQ1 | DEAD-box helicase 3 X-linked | DDX3X | 0.411 |
| A0A288CG57 | Eukaryotic translation elongation factor 1 alpha 1 | EEF1A1 | 0.403 |
| A0A480V1M7 | Adaptor related protein complex 2 subunit beta 1 | AP2B1 | 0.395 |
| A0A5G2RGI4 | Ubiquitin conjugating enzyme E2 N like | UBE2NL | 0.394 |
| A0A287A4Y2 | Guanosine monophosphate reductase | GMPR | 0.370 |
| Q6QA25 | Tropomyosin 3 | TPM3 | 0.349 |
| A0A4X1TI48 | Nucleolin | NCL | 0.317 |
| A0A5G2QL81 | Proteasome 20s subunit alpha 4 | PSMA4 | 0.313 |
| M3VJZ7 | LIM and SH3 protein 1 | LASP1 | 0.283 |
| A0A4X1UM41 | Tyrosine 3-monooxygenase/tryptophan 5-monooxygenase activation protein gamma | YWHAG | 0.260 |
| A0A287AWI9 | Eukaryotic translation elongation factor 2 | EEF2 | 0.206 |
| A0A5G2QLY5 | Staphylococcal nuclease domain-containing protein | SND1 | 0.205 |
| F1RWJ5 | Karyopherin subunit beta 1 | KPNB1 | 0.197 |
| F2Z5L7 | Proteasome 20s subunit alpha 1 | PSMA1 | 0.196 |
| H6UWK6 | Olfactomedin like 3 | OLFML3 | -0.288 |
| K9J6K4 | Leukotriene A4 hydrolase | LTA4H | -0.325 |
| F1RGJ3 | Heat shock protein family A 70kDa protein 9 | HSPA9 | -0.336 |
| A0A480SDM7 | Kinectin 1 | KTN1 | -0.345 |
| A0A5G2RGL7 | Malate dehydrogenase 2 | MDH2 | -0.373 |
| A5A8V6 | Heat shock 70kDa protein 1A | HSPA1A | -0.374 |
| A0A4X1VY68 | NADH-cytochrome b5 reductase | LOC100524254 | -0.382 |
| A0A480W9F9 | Serpin family G member 1 | SERPING1 | -0.394 |
| A5A8V8 | U6 snRNA-associated Sm-like protein LSm2 | LSM2 | -0.396 |
| A0A287AJQ2 | Phosphoglycerate mutase 1 | PGAM1 | -0.401 |
| A0A5G2RFW9 | Mesencephalic astrocyte derived neurotrophic factor | MANF | -0.402 |
| A0A4X1W115 | Small nuclear ribonucleoprotein Sm D2 (Sm-D2) | SNRPD2 | -0.408 |
| G9F6X9 | Dihydropyrimidinase-like 2 | DPYSL2 | -0.412 |
| F1S710 | Calcyclin binding protein | CACYBP | -0.416 |
| A0A481C7A2 | Collagen alpha-1(XVIII) chain isoform 2 | COL18 | -0.436 |
| A0A4X1UGZ2 | Biliverdin reductase A | BLVRA | -0.470 |
| A0A5G2RN04 | ADP ribosylation factor like GTPase 8B | ARL8B | -0.487 |
| A0A287BP50 | Thioredoxin like 1 | TXNL1 | -0.489 |
| A0A4X1VD81 | Eukaryotic translation initiation factor 3 subunit J | EIF3J | -0.498 |
| F2Z5C1 | Annexin A5 | ANXA5 | -0.521 |
| Q27HV0 | UDP-N-acetylglucosamine--peptide N-acetylglucosaminyltransferase | OGT | -0.538 |
| A0A5G2R6J1 | Proteasome 20s subunit beta 2 | PSMB2 | -0.540 |
| A0A481AUZ6 | Zyxin | ZYX | -0.557 |
| A0A480TTD3 | Importin-5 | IPO5 | -0.558 |
| A0A5G2QFC2 | Malate dehydrogenase 1 | MDH1 | -0.573 |
| F1S3E0 | Transmembrane emp24 domain-containing protein 9 | TMED9 | -0.589 |
| A0A480X877 | Transforming growth factor beta induced | TGFBI | -0.592 |
| A0A4X1SGU8 | NAPDH:Adrenodoxin reductase |  | -0.598 |
| A0A4X1UU78 | Aminoacyl tRNA synthetase complex interacting multifunctional protein 1 | AIMP1 | -0.614 |
| A0A286ZSA7 | 3-hydroxybutyrate dehydrogenase 2 | BDH2 | -0.642 |
| F1SQ09 | Lumican | LUM | -0.682 |
| A0A4X1UED9 | Heat shock protein family A 70kDa member 2 | HSPA2 | -0.682 |
| A0A4X1V1Z6 | Aspartate beta-hydroxylase | ASPH | -0.706 |
| A0A287BL58 | PBX homeobox interacting protein 1 | PBXIP1 | -0.721 |
| A0A480TAI8 | Caldesmon 1 | CALD1 | -0.730 |
| K9J6I5 | Endoplasmic reticulum aminopeptidase 1 | ERAP1 | -0.731 |
| P79382 | Microsomal glutathione S-transferase 1 | MGST1 | -0.732 |
| A0A4X1W2W9 | NSF attachment protein alpha | NAPA | -0.735 |
| A0A4X1SD76 | Prune exopolyphosphatase 1 | PRUNE1 | -0.744 |
| A0A4X1W7W4 | Nidogen 2 | NID2 | -0.754 |
| A0A4X1SGX4 | Serpin family A member 6 | SERPINA6 | -0.773 |
| A0A4X1UVY8 | Poly(U)-binding-splicing factor 60 | PUF60 | -0.780 |
| A0A481BC81 | Ribose-5-phosphate isomerase | RPIA | -0.783 |
| A0A5G2QQE9 | Collagen type I alpha 1 chain | COL1A1 | -0.796 |
| A0A286ZV95 | Transmembrane emp24 domain-containing protein 10 | TMED10 | -0.856 |
| A0A4X1V152 | Zinc finger DBF-type containing 2 | ZDBF2 | -0.865 |
| D0G0C9 | Chaperonin containing TCP1 subunit 7 | CCT7 | -0.887 |
| A0A5G2R0E8 | Mannosidase alpha class 2B member 1 | MAN2B1 | -0.942 |
| A0A4X1SH59 | SEC61 translocon subunit beta | SEC61B | -0.946 |
| I3LDS3 | Keratin 10 | KRT10 | -1.009 |
| A0A286ZPD7 | Transmembrane emp24 domain-containing protein 7 | TMED7 | -1.073 |
| A0A4X1SME4 | Pre-mRNA processing factor 4B | PRPF4B | -1.082 |
| I3LS60 | NAD(P)HX dehydratase | NAXD | -1.101 |
| A0A4X1TJN9 | Copper chaperone for superoxide dismutase | CCS | -1.195 |
| F1RG16 | Heterogeneous nuclear ribonucleoprotein F | HNRNPF | -1.202 |
| A0A287A2Q7 | Collagen type II alpha 1 chain | COL2A1 | -1.499 |
| A0A4X1V1E0 | Protein phosphatase, Mg2+/Mn2+ dependent 1F | PPM1F | -1.791 |
| L8B0U8 | IgG heavy chain | IGHG | -1.901 |
| A0A286ZLL9 | Synaptopodin | SYNPO | -2.252 |
| A0A4X1VSE7 | Cytoskeleton associated protein 5 | CKAP5 | -2.390 |
| A0A4X1VD60 | Protein phosphatase 2 regulatory subunit alpha | PPP2R2A | -2.708 |

**log2(FC) = Log two-fold change in HZ relative to HC gilt ovaries**
